# Unveiling the molecular complexity of proliferative diabetic retinopathy through scRNA-seq, AlphaFold 2, and machine learning

**DOI:** 10.3389/fendo.2024.1382896

**Published:** 2024-05-10

**Authors:** Jun Wang, Hongyan Sun, Lisha Mou, Ying Lu, Zijing Wu, Zuhui Pu, Ming-ming Yang

**Affiliations:** ^1^ Department of Endocrinology, Shenzhen People’s Hospital (The Second Clinical Medical College of Jinan University; The First Affiliated Hospital, Southern University of Science and Technology), Shenzhen, China; ^2^ Department of Ophthalmology, Shenzhen People’s Hospital (The Second Clinical Medical College, Jinan University; The First Affiliated Hospital, Southern University of Science and Technology), Shenzhen, China; ^3^ Imaging Department, Shenzhen Institute of Translational Medicine, The First Affiliated Hospital of Shenzhen University, Shenzhen Second People’s Hospital, Shenzhen, China; ^4^ MetaLife Center, Shenzhen Institute of Translational Medicine, Guangdong, Shenzhen, China

**Keywords:** diabetic retinopathy, single-cell analysis, oxidative stress, AlphaFold 2, NMF, PPI, machine learning, ALKBH1

## Abstract

**Background:**

Proliferative diabetic retinopathy (PDR), a major cause of blindness, is characterized by complex pathogenesis. This study integrates single-cell RNA sequencing (scRNA-seq), Non-negative Matrix Factorization (NMF), machine learning, and AlphaFold 2 methods to explore the molecular level of PDR.

**Methods:**

We analyzed scRNA-seq data from PDR patients and healthy controls to identify distinct cellular subtypes and gene expression patterns. NMF was used to define specific transcriptional programs in PDR. The oxidative stress-related genes (ORGs) identified within Meta-Program 1 were utilized to construct a predictive model using twelve machine learning algorithms. Furthermore, we employed AlphaFold 2 for the prediction of protein structures, complementing this with molecular docking to validate the structural foundation of potential therapeutic targets. We also analyzed protein−protein interaction (PPI) networks and the interplay among key ORGs.

**Results:**

Our scRNA-seq analysis revealed five major cell types and 14 subcell types in PDR patients, with significant differences in gene expression compared to those in controls. We identified three key meta-programs underscoring the role of microglia in the pathogenesis of PDR. Three critical ORGs (ALKBH1, PSIP1, and ATP13A2) were identified, with the best-performing predictive model demonstrating high accuracy (AUC of 0.989 in the training cohort and 0.833 in the validation cohort). Moreover, AlphaFold 2 predictions combined with molecular docking revealed that resveratrol has a strong affinity for ALKBH1, indicating its potential as a targeted therapeutic agent. PPI network analysis, revealed a complex network of interactions among the hub ORGs and other genes, suggesting a collective role in PDR pathogenesis.

**Conclusion:**

This study provides insights into the cellular and molecular aspects of PDR, identifying potential biomarkers and therapeutic targets using advanced technological approaches.

## Introduction

Proliferative diabetic retinopathy (PDR), an advanced stage of diabetic retinopathy, is a leading cause of irreversible blindness in the productive-age population worldwide (1, 2). Characterized by retinal neovascularization leading to severe complications such as neovascular glaucoma, vitreous hemorrhage, and retinal detachment, the pathogenesis of PDR has not been fully elucidated (3, 4). Despite recent advances in imaging and management (5), understanding the underlying molecular mechanisms is crucial for developing effective therapies.

Oxidative stress, which is notably exacerbated in diabetes, plays a pivotal role in PDR pathogenesis (6). It damages mitochondrial structures and DNA in the retinal vasculature, impairing cellular function (7). This stress is a key contributor to neovascular unit insults, underpinning the core pathophysiology of PDR. Additionally, diabetic patients are more susceptible to oxidative stress due to impaired defense mechanisms, further emphasizing the role of oxidative stress in the development and progression of diabetic retinopathy, including PDR (8).

Single-cell RNA sequencing (scRNA-seq) has significantly advanced disease research by providing detailed insights into the cellular and molecular dimensions of various diseases (9, 10). Its ability to dissect gene expression at the individual cell level reveals the intricate cellular landscape of PDR, distinguishing between diseased and healthy states (11). The study carried out by Hu et al. provides valuable insights into the use of scRNA-seq in studying PDR (12). These authors highlighted the application of scRNA-seq for gene expression profiling, identifying cell populations in fibrovascular membranes from PDR patients, and revealing the novel role of microglia in the fibrovascular membrane of PDR. These studies collectively emphasize the significance of scRNA-seq in unraveling the molecular and cellular complexities of PDR, offering a promising approach for further research and potential therapeutic interventions.

Concurrently, the integration of machine learning algorithms, particularly in predictive modeling, has introduced a new dimension to biomedical research (13, 14). These algorithms, including LASSO, Ridge, and Elastic Net, facilitate the development of predictive models for PDR, thereby increasing the accuracy of diagnoses and informing personalized treatment approaches.

In our study, we combined single-cell sequencing with advanced machine learning methods, as well as Non-negative Matrix Factorization (NMF), to uncover transcriptional and oxidative stress signatures in PDR. Our goal was to pinpoint oxidative stress-related genes (ORGs) that could serve as biomarkers, aiming to enhance the diagnostic and therapeutic landscape of PDR.

## Methods

### Data processing

ScRNA-seq data from five proliferative diabetic retinopathy (PDR) patients (GSE165784) (12) and three control samples (15) were processed alongside two bulk RNA PDR patient cohorts from the GEO database (cohort 1: GSE160306 (16), 76 samples; cohort 2: GSE102485 (17), 25 samples). Oxidative stress-related genes (ORGs) were identified from the Gene Ontology and PathCards databases.

### Single-cell data analysis of PDR patients

The single-cell data of five PDR patients (12) and three healthy controls (15) were analyzed via Seurat (18). We filtered cells based on mitochondrial content (<10%), cell count (>300), and gene number (1000-5000). The t-distributed stochastic neighbor embedding (t-SNE) (19) and ‘RunHarmony’ functions (20) facilitated visualization and batch effect correction. Cell subtypes were annotated according to cell markers from the original study (12, 15). In the differential expression analysis between microglia and mesenchymal cells in PDR versus control samples, the mitochondrial and ribosomal genes were removed. We used the Wilcoxon signed-rank test to identify significant genes (adjusted P value <0.05, absolute log_2_FC >1).

### Non-negative matrix factorization and meta-program detection of microglia in PDR patients

NMF analysis, specifically consensus NMF (cNMF), was applied to microglia in PDR samples, standardizing negative values to zero. After more than 100 iterations, we explored the components (k) ranging from 2 to 10 signatures, determining the optimal component number via a diagnostic plot from the provided tutorial (https://github.com/dylkot/cNMF) (21). A two-step gene ranking algorithm was used to identify nonoverlapping gene modules, which were further analyzed for expression patterns using Pearson correlations and hierarchical clustering, revealing three distinct meta-programs.

### Establishment of a machine learning-driven predictive ORG model for PDR patients

Twelve machine learning algorithms, including (1) Least Absolute Shrinkage and Selection Operator (LASSO), (2) Ridge, (3) Elastic network (Enet), (4) Stepglm, (5) Support Vector Machines (SVM), (6) GlmBoost, (7) Linear Discriminant Analysis (LDA), (8) Partial Least Squares Regression for Generalized Linear Models (plsRglm), (9) Random Forest (RSF), (10) Generalized Boosted Regression Models (GBMs), (11) XGBoost, (12) Naive Bayes, were utilized to develop a predictive ORG model for PDR. We constructed 109 model combinations, trained initial models with the GSE160306 cohort and validated them with the GSE102485 cohort. Model performance was assessed using the AUC.

### Prediction of the structure of proteins

We utilized AlphaFold 2, a tool that has achieved remarkably accurate levels comparable to those obtained through human observation via advanced techniques such as cryoelectron microscopy, for the prediction of protein structures (22). For our specific study objectives, we used AlphaFold 2 to predict the structures of select proteins relevant to our research. We focused on the proteins ALKBH1, PSIP1, and ATP13A2, which play significant roles in the context of PDR. The sequences of these proteins were meticulously retrieved from the NCBI database (23).

### Molecular docking analysis

To investigate the binding affinities and interaction patterns of the drug candidates with their targets, we utilized AutoDock Vina 1.2.2, a software designed for in silico protein–ligand docking (24). The molecular structure of resveratrol was obtained from the PubChem Compound database (https://pubchem.ncbi.nlm.nih.gov/) (25). AlphaFold 2 was used to generate the 3D coordinates for ALKBH1. Before docking analysis, all proteins and ligand files were prepared by converting them into PDBQT format. This preparation involved the removal of water molecules and the addition of polar hydrogen atoms to ensure accurate docking simulations. The docking grid box was strategically positioned to encompass the target protein’s domain, allowing for unhindered molecular movement within the simulation. The dimensions of the grid box were set to 30 Å × 30 Å × 30 Å, with a grid point spacing of 0.05 nm to capture detailed interaction data. The molecular docking studies were conducted using AutoDock Vina 1.2.2 (http://autodock.scripps.edu/).

### Protein interaction network analysis of key ORGs

In our study, we investigated protein interactions involving three pivotal ORGs. The use of the STRING database (https://string-db.org/) (26), a comprehensive resource, enabled us to compile and amalgamate data on protein−protein interactions (PPIs). Our focus was directed toward interactions with confidence scores surpassing 0.7, a threshold set to ensure the biological relevance and significance of these interactions.

To deepen our analysis and improve its visualization, we transferred the relevant data into Cytoscape (version 3.8.2) (27). Within the Cytoscape environment, we leveraged the capabilities of the cytoHubba plugin. This allowed us to pinpoint and rank the top 10 nodes in the PPI network. The ranking process utilized seven distinct algorithms, each contributing a unique perspective to the analysis. These algorithms included the following: Radiality, which measures the centrality of a node; Maximum Neighborhood Component (MNC), which assesses the largest connected component around a node; Maximum Clique Centrality (MCC), which focuses on the largest clique a node belongs to; Edge Percolated Component (EPC), which evaluates the connectivity and clustering; DMNC, which is the Maximum Neighborhood Component Centrality, a derivative of the MNC; Degree, which counts the number of edges linked to a node; and Closeness, which measures the average distance to other nodes. To synthesize and present our findings, we utilized an UpSet diagram.

### Identification of hub genes associated with PDR

To identify the hub genes associated with PDR, we used the Comparative Toxicogenomics Database (CTD, http://ctdbase.org/) (28). Utilizing the CTD, we conducted an in-depth analysis to unravel the connections between potential key genes and a spectrum of relevant conditions. This included not only PDR but also a broader scope of related health issues, such as other eye diseases, retinal disorders, vascular diseases, complications arising from diabetes, and diabetes mellitus itself.

### Statistical analysis

All the statistical analyses of single cells were performed with R (version 4.3.1). A *P* value less than 0.05 was considered to indicate statistical significance if not explicitly stated.

## Results

### Analysis of single-cell RNA sequencing data

In this study, we conducted an in-depth analysis of single-cell RNA sequencing data from five patients with proliferative diabetic retinopathy (PDR) (GSE165784) (12) and three healthy controls (15), implementing t-distributed stochastic neighbor embedding (t-SNE) for visualization post-quality control and data normalization. This approach effectively distinguished between cellular clusters of the PDR and control cohorts.

**Figure 1:**
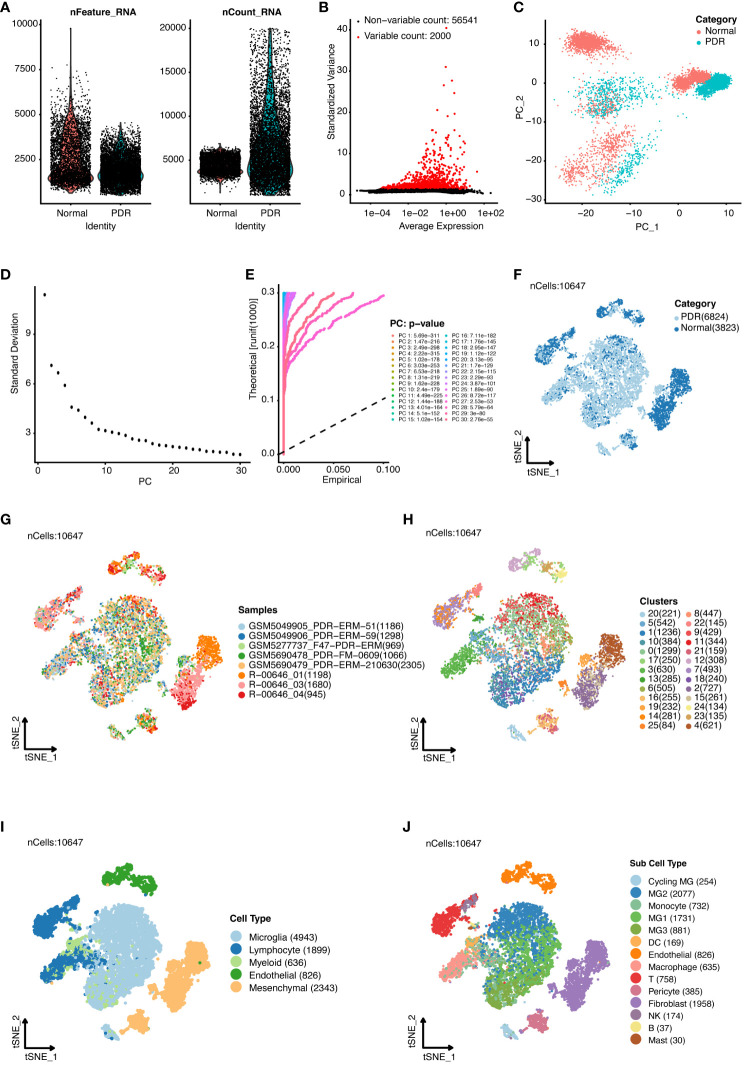
Single-cell RNA sequencing analysis of proliferative diabetic retinopathy (PDR) samples compared with normal samples. **(A)** Quality control of single-cell RNA sequencing data for PDR and normal samples. **(B)** Identification of highly variable genes. The top 2000 variable genes are shown as red dots. **(C)** Principal component analysis. Accordingly, we classified the cell groups into two categories. ElbowPlot **(D)** and JackStrawPlot **(E)** of principal components. T-distributed stochastic neighbor embedding (t-SNE) analysis of different groups **(F)**, 8 samples **(G)**, 26 clusters **(H)**, five major cell types **(I)**, and 14 subcell types **(J)**.

**Figure 1A** displays the range and individual RNA counts per cell, reflecting successful quality control measures for our sample analysis. We highlighted the 2000 genes with the highest variability across samples in **Figure 1B**. To further dissect this complexity, we applied linear dimensionality reduction to compute principal components (PCs), as illustrated in **Figure 1C**. The determination of significant PCs was aided by the integration of both ElbowPlot (**Figure 1D**) and JackStrawPlot (**Figure 1E**), setting the stage for more nuanced analyses. The distribution of cells across the PDR and control groups is presented in **Figure 1F**, with the study encompassing 5 PDR and 3 control samples, as depicted in **Figure 1G**. A total of 26 clusters were identified across the samples (**Figure 1H**). Through marker analysis from the original study (12), we classified cells into five primary types: microglia, lymphocytes, myeloid cells, endothelial cells, and mesenchymal cells (**Figure 1I**). This categorization was further refined, resulting in the identification of 14 distinct subcell types (**Figure 1J**).

### Analysis of gene expression variations and cell-cell interactions in PDR

**Figure 2:**
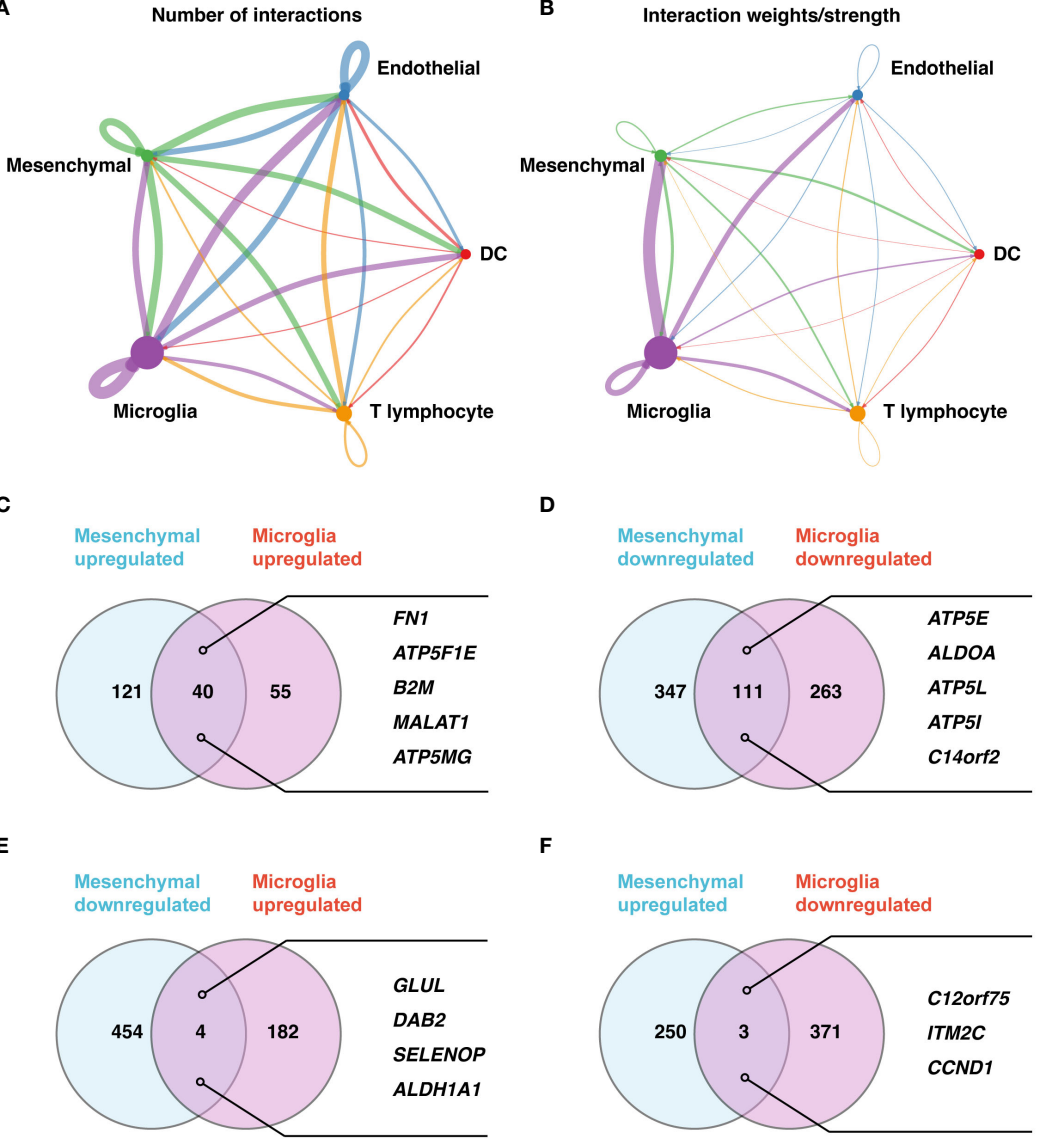
Detailed analysis of cell-cell communication and gene expression in PDR. **(A, B)** Cell-cell communication network maps for five major cell types based on the number of involved genes **(A)** and interaction weights/strengths **(B)**. **(C–F)** Gene expression analysis of microglia and mesenchymal cells. Upregulated **(C)** and downregulated **(D)** genes in both cell types. **(E)** Downregulated genes in mesenchymal cells but upregulated genes in microglia. **(F)** Upregulated genes in mesenchymal cells but downregulated genes in microglia.

Our investigation of differential gene expression and intercellular communication within the retinal microenvironment of PDR patients highlighted important findings. We observed pronounced ligand-receptor interactions among various cell types, with notable interactions between microglia and mesenchymal cells, as well as between microglia and endothelial cells (**Figures 2A, B**). These interactions shed light on the intricate signaling pathways that could be instrumental in the development and progression of PDR, suggesting potential therapeutic targets.

In a detailed analysis of gene expression between microglia and mesenchymal cells in PDR versus control samples, 40 genes were upregulated, and 111 genes were downregulated in both cell types (**Figures 2C, D**; **Supplementary Tables 1**–**4**). The upregulated genes, including FN1, ATP5F1E, B2M, MALAT1, and ATP5MG, and downregulated genes, such as ATP5E, ALDOA, ATP5L, ATP5I, and C14orf2, indicate a complex regulatory landscape. Furthermore, we revealed nuanced gene expression patterns: GLUL, DAB2, SELENOP, and ALDH1A1 were downregulated in mesenchymal cells but upregulated in microglia (**Figure 2E**; **Supplementary Tables 5**, **6**), while C12orf75, ITM2C, and CCND1 showed the opposite pattern (**Figure 2F**; **Supplementary Tables 7**, **8**).

### Identification of transcriptional programs in PDR microglia cells using non-negative matrix factorization

In our detailed investigation of specific microglia within PDR samples, we employed the sophisticated technique of NMF to determine the unique transcriptional landscape of these cells. This advanced approach allowed us to systematically catalog various gene modules, which are fundamentally crucial in defining the distinct states of cells. Through this meticulous process, we were able to identify and analyze patterns of gene coexpression within individual PDR samples.

Our comparative analysis across multiple PDR samples was instrumental in revealing recurring gene modules. This aspect of our study was particularly significant because it effectively minimized the impact of technical variations, thereby enhancing the reliability and accuracy of our findings. By focusing on these gene modules, we gained valuable insights into the transcriptional intricacies inherent in PDR microglia.

**Figure 3:**
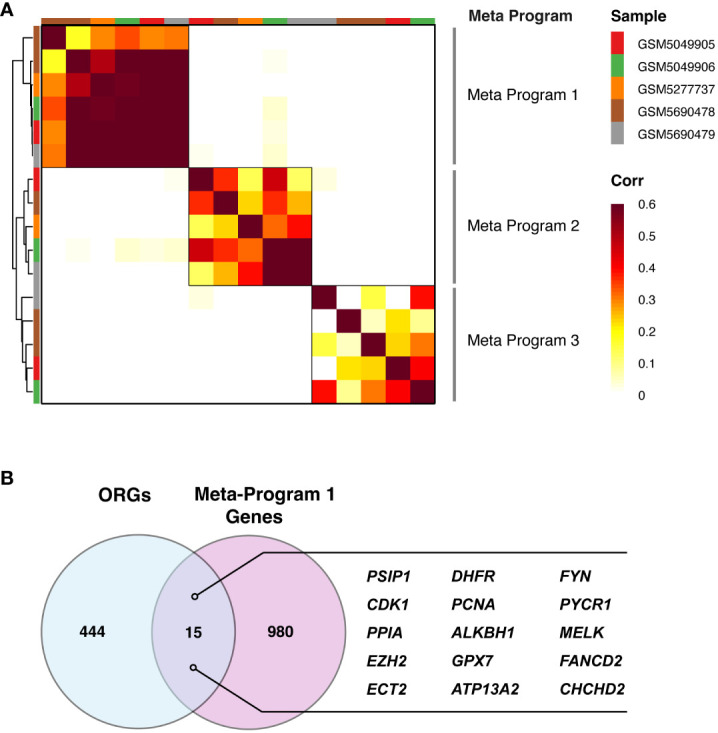
Catalog of PDR gene modules in microglia. **(A)** Heatmap demonstrating the significance of the overlap between PDR gene modules in microglia, identifying three consensus modules: Meta-Program 1, Meta-Program 2, and Meta-Program 3. **(B)** Identification of 15 genes at the intersection of Meta-Program 1 and oxidative stress-related genes (ORGs) from the Gene Ontology and PathCards databases.

One of the most noteworthy outcomes of our analysis was the identification of three distinct meta-programs. These meta-programs were discerned and clustered based on their correlation coefficients, providing a clear representation of the transcriptional synergy within microglia. The top-scoring genes of these meta-programs were characterized, as depicted in **Figure 3A**. Notably, Meta-Program 1 emerged as particularly prominent, exhibiting the highest level of correlation among the three. These results suggest that the genes within Meta-Program 1 are potentially central to the transcriptional identity and function of microglia in the context of PDR.

Elucidation of these meta-programs is important to our understanding of PDR. This study provides a novel perspective on the transcriptional dynamics of microglia, a critical component of disease pathology. This insight not only enhances our understanding of the molecular mechanisms underlying PDR but also opens up new directions for targeted therapeutic strategies aimed at modulating these specific transcriptional programs.

### Development and validation of the oxidative stress-related gene predictive model for PDR

**Figure 4:**
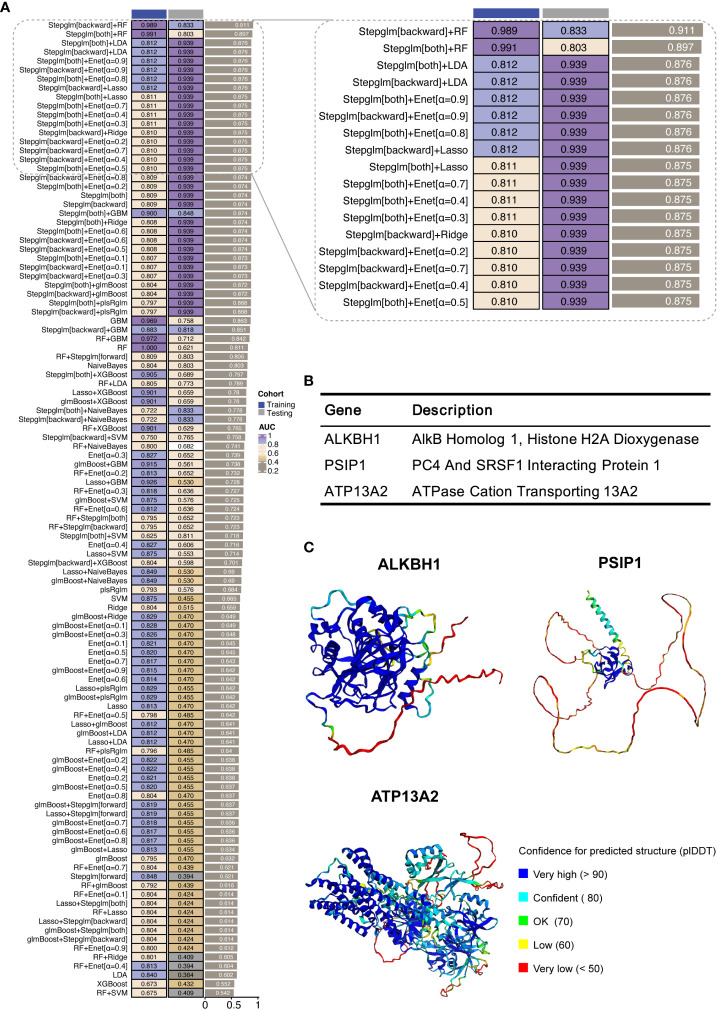
Development of machine learning-derived predictive models. **(A)** AUC results for combinations of machine learning algorithms in the training and validation cohorts. The training cohort was GSE160306, and the validation cohort was GSE102485. **(B)** Description of the three hub ORGs included in the highest-performing model. **(C)** Protein structures of three hub ORGs predicted using AlphaFold 2.

To develop a predictive model for PDR based on ORGs, we examined a subset of 15 genes that notably intersected within Meta-Program 1, as illustrated in **Figure 3B**. We constructed ORG models by twelve diverse machine learning algorithms, including (1) Least Absolute Shrinkage and Selection Operator (LASSO), (2) Ridge, (3) Elastic network (Enet), (4) Stepglm, (5) Support Vector Machines (SVM), (6) GlmBoost, (7) Linear Discriminant Analysis (LDA), (8) Partial Least Squares Regression for Generalized Linear Models (plsRglm), (9) Random Forest (RSF), (10) Generalized Boosted Regression Models (GBMs), (11) XGBoost, (12) Naive Bayes (**Figure 4A**). Among the 109 models constructed, the cream of the crop emerged in the form of models based on a sophisticated Stepglm [backward]+RF approach. These standout models prominently featured three key ORGs, ALKBH1, PSIP1, and ATP13A2, as delineated in **Figure 4B**. The importance of this model was unmistakably demonstrated in the training cohort (GSE160306), in which an outstanding area under the curve (AUC) of 0.989 was achieved. This exceptional level of predictive accuracy underlines the model’s formidable potential as a tool for diagnosing PDR.

To further validate the model’s applicability in a clinical setting, we undertook a validation study using an external cohort (GSE102485). The results were encouraging, as the model retained a significant level of diagnostic accuracy, as evidenced by an AUC of 0.833. This performance in an external cohort not only reinforces the model’s robustness but also underscores its potential utility as an early detection and ongoing monitoring tool for PDR.

### Structural prediction and molecular docking analysis

In our study, we harnessed the ability of AlphaFold 2 technology to predict the complex structures of three pivotal ORGs, ALKBH1, PSIP1, and ATP13A2, as shown in **Figure 4C**. The predictive confidence for ALKBH1 and ATP13A2 was notably high, whereas PSIP1 demonstrated lower confidence levels and was subsequently not included in further analysis.

**Figure 5:**
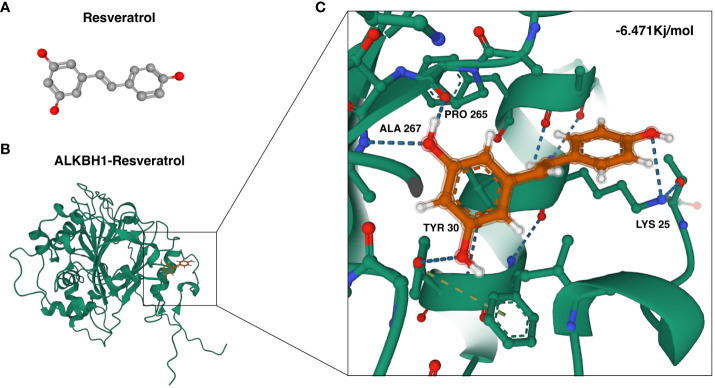
Molecular docking of resveratrol with ALKBH1. **(A)** Three-dimensional configuration of the ALKBH1 protein. **(B)** Illustration of the binding interaction between the ALKBH1 protein and resveratrol. **(C)** A closer view of the molecular docking of resveratrol with ALKBH1, highlighting local amplification details.

Furthermore, to assess the binding affinity of potential therapeutic agents for these targets, we conducted a molecular docking analysis. Specifically, we explored the interaction between ALKBH1 and the candidate drug resveratrol utilizing AutoDock Vina v.1.1.2 for this purpose. The analysis provided insights into the binding modes and calculated the binding energies for the interactions (**Figures 5A–C**). The derived binding energy for the ALKBH1-resveratrol complex was -6.471 kcal/mol, suggesting a highly stable interaction. This strong and stable binding affinity further underscores the potential therapeutic relevance of targeting ALKBH1 with resveratrol in the context of oxidative stress-related conditions.

### Protein interaction analysis of key ORGs in PDR

Next, we explored the protein-protein interactions (PPIs) of these ORGs. For this purpose, we utilized the STRING database, which is renowned for its extensive protein interaction data. Our focus was on interactions with confidence scores exceeding 0.7, ensuring that only biologically significant and reliable interactions were considered. This selective approach was instrumental in sifting through vast data to identify meaningful connections that could be crucial in the context of PDR.

**Figure 6:**
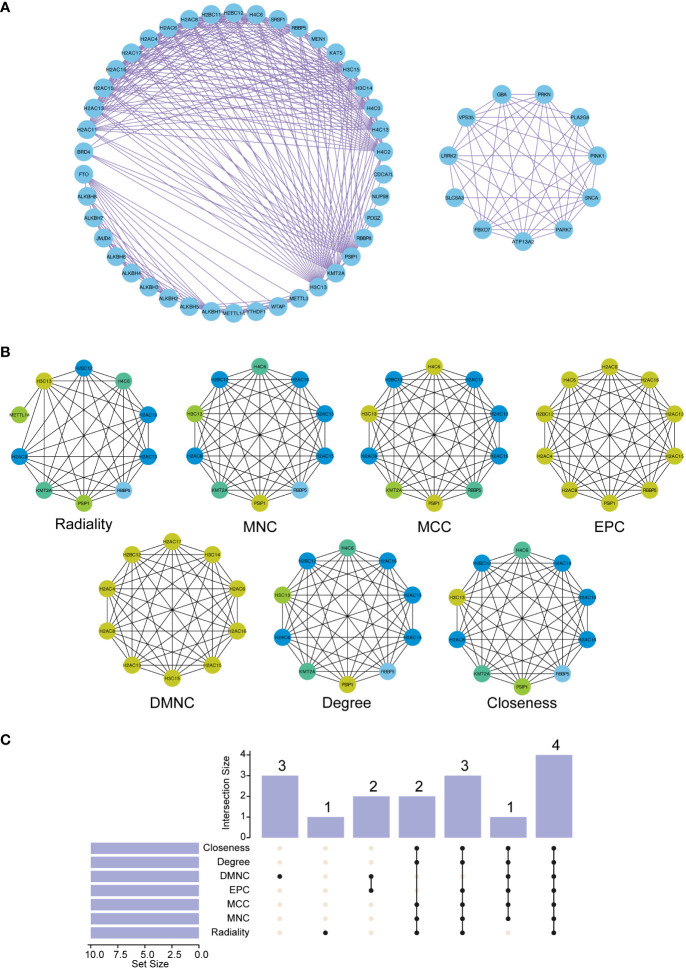
Construction of the protein-protein interaction (PPI) network and functional enrichment analysis of the three hub ORGs. **(A)** The PPI network was constructed based on 50 genes closely related to the three hub ORGs. **(B)** The top ten hub genes in the PPI network were identified using seven analytical algorithms. **(C)** UpSet plot displaying overlapping genes identified by all algorithms.

The PPI network enriched with these curated data was then intricately analyzed using Cytoscape. This platform enabled us to visualize and dissect the complex web of interactions. Using the cytoHubba plugin within Cytoscape, we systematically identified the top 10 nodes in the network utilizing a suite of seven sophisticated algorithms. These included Radiality, Maximum Neighborhood Component (MNC), Maximum Clique Centrality (MCC), EPC (Edge Percolated Component), DMNC (Maximum Neighborhood Component Centrality), Degree, and Closeness, each offering a unique lens to view and understand the network’s structure. **Figures 6A, B** depict these findings, revealing a comprehensive map of the interactions.

Furthermore, to emphasize the interconnected nature of these interactions, we constructed an UpSet diagram (**Figure 6C**). This visualization succinctly highlighted the convergence of hub genes across different algorithms, revealing key proteins such as H2AC8, H2BC12, H2AC13, and H2AC16 that were consistently central across all algorithms, as detailed in **Supplementary Table 9**. This representation was instrumental in highlighting the core genes within the network, thereby elucidating their potential collective role in the pathophysiology of PDR.

### Integrating comparative toxicogenomics database analysis with PDR research

**Figure 7:**
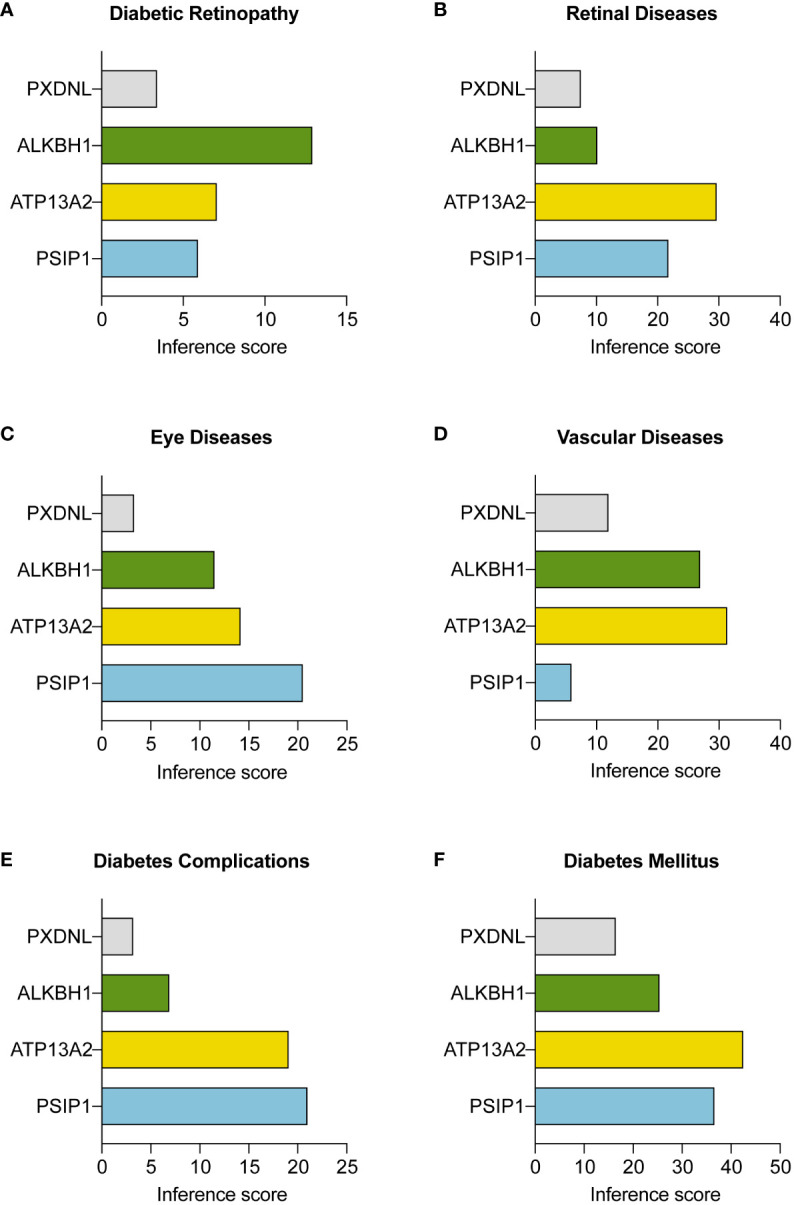
Interactions between three key ORGs and the negative control gene PXDNL across various disease conditions. The analysis was conducted with the Comparative Toxicogenomics Database (CTD; http://ctdbase.org/). The inference scores between the three hub ORGs and **(A)** diabetic retinopathy, **(B)** retinal diseases, **(C)** eye diseases, **(D)** vascular diseases, **(E)** diabetes complications, and **(F)** diabetes mellitus are shown in bar plots.

To complement our protein interaction analysis, we utilized CTD as an instrumental resource. The CTD facilitated the expansion of our study to investigate the connections between our identified hub ORGs and a range of conditions associated with PDR, such as diabetic retinopathy, various eye and retinal diseases, vascular complications, and diabetes mellitus itself. **Figures 7A–F** display these connections, emphasizing the marked correlation between genes ALKBH1, PSIP1, ATP13A2, and the aforementioned conditions, validated by substantial reasoning scores within the CTD. Additionally, we incorporated a “negative control” gene, PXDNL, an unrelated ORG, to bolster the conclusiveness of our analysis.

## Discussion

Proliferative diabetic retinopathy (PDR) poses a significant challenge in diabetes management and often leads to irreversible blindness. Current treatments such as panretinal photocoagulation have limitations, including potential adverse effects on visual acuity (29). Novel approaches such as CD40-TRAF6 inhibition (30) and anti-IL17A therapy (31) show promise in mouse models but require further clinical validation. These limitations underscore the pressing need for more effective and precise therapeutic strategies.

In our study, the integration of single-cell sequencing and Non-negative Matrix Factorization (NMF) was pivotal in revolutionizing our understanding of the transcriptional intricacies in PDR. This advanced methodological approach facilitated in-depth analysis of the disease transcriptional landscape, revealing the existence of specific gene modules and delineating three crucial meta-programs.

Our focused analysis, through the lens of NMF, allowed us to dissect the intricate patterns of gene expression, revealing how different gene modules interact and contribute to the pathophysiology of PDR. This nuanced understanding of gene modules and their interplay is critical, as it sheds light on the underlying mechanisms that drive the disease. In particular, the discovery of oxidative stress-related genes (ORGs), which are key players within these meta-programs, has been illuminating. This highlights the significant role that oxidative stress, a known factor in diabetic complications, plays in the progression of PDR.

In the context of oxidative stress and its implications for disease pathogenesis, the identification and study of ALKBH enzymes, particularly ALKBH8, have been pivotal. Previous research has elucidated the role of these enzymes in the intricate regulation of reactive oxygen species (ROS) production and oxidative stress, which are crucial processes in cellular homeostasis and disease development. For example, studies have highlighted the role of ALKBH8 in the development of human bladder cancer, where it contributes to the disease process by downregulating NAD(P)H oxidase-1 (NOX-1) and subsequently activating pathways such as the c-jun NH2-terminal kinase (JNK) and p38 pathways, which are involved in NADPH oxidase 1-dependent ROS production and apoptosis induction (32). Additionally, ALKBH8 has been implicated in the reduction of ROS production through similar mechanisms and in the regulation of selenocysteine protein expression, which serves as a defense against ROS damage in response to oxidative stress (33). These findings collectively underscore the substantial role of ALKBH, particularly ALKBH8, in the regulation of oxidative stress and its relevance to various disease processes.

Similarly, ATP13A2 has been extensively studied for its role in the regulation of cellular responses to oxidative stress. This gene is implicated in protective mechanisms against mitochondrial toxins such as rotenone, which is an environmental risk factor for Parkinson’s disease (34). The function of ATP13A2 in mitigating oxidative stress is multifaceted. PSP not only aids in reducing levels of intracellular oxidative damage but also enhances the clearance of oxidatively damaged macromolecules (35). This finding suggested that ATP13A2 plays a significant protective role against oxidative stress, underscoring its importance in maintaining cellular health and preventing damage. Furthermore, the impaired function of ATP13A2 has been directly linked to increased oxidative stress in human neuroblastoma cells, highlighting its critical role in cellular defense mechanisms against oxidative damage (36).

These insights into ALKBH and ATP13A2 provide a deeper understanding of the molecular mechanisms by which oxidative stress influences disease progression and pathology. The significant relationship of these genes with the regulation of oxidative stress emphasizes their potential as therapeutic targets. In the context of PDR, where oxidative stress plays a central role, understanding these mechanisms is crucial. This approach opens potential avenues for targeted therapies that modulate oxidative stress pathways, potentially offering more effective treatment options for conditions such as PDR and beyond.

Therefore, elucidating the roles of ALKBH and ATP13A2 in oxidative stress regulation not only enhances our understanding of the cellular response to oxidative challenges but also positions these genes as key players in the development of novel therapeutic strategies for diseases where oxidative stress is a contributing factor.

Incorporating the AlphaFold 2 technology (22) into our research represents an innovation in our study. AlphaFold 2, an advanced protein structure prediction tool developed by DeepMind, has revolutionized the field of structural biology. Its ability to predict protein structures with unprecedented accuracy provides invaluable insights into the functional mechanisms of proteins at the molecular level.

In the context of our study on PDR, the application of AlphaFold 2 allowed us to predict the structures of key ORGs, namely, ALKBH1, PSIP1, and ATP13A2. This capability is crucial because it provides a deeper understanding of protein configurations and their potential interactions, which are often pivotal in determining their functional roles in cellular processes. The structural insights gained from AlphaFold 2 significantly augmented our understanding of protein−protein interactions (PPIs) and the molecular pathways in which these ORGs are involved. The ability to visualize the precise structure of these proteins aids in elucidating their functional domains, interaction sites, and potential binding mechanisms, which are essential for elucidating their roles in the pathogenesis of PDR. Furthermore, the application of the AlphaFold 2 in our study sets a precedent for future research on diabetic retinopathy and other related diseases. By enabling a more accurate prediction of protein structures, new possibilities are available for the development of targeted therapeutic interventions, as structural insights are crucial for drug design and discovery.

Our findings resonate with and build upon existing related research in the field, such as the notable work of Hu et al., which focused on the involvement of microglia in PDR (12). This alignment with the findings of previous studies not only validates our findings but also adds a new dimension to our collective understanding of the disease. By contextualizing our results within the broader scientific narrative, we underscore the importance of oxidative stress in PDR pathogenesis and open potential avenues for targeted therapeutic interventions.

There are limitations to this study. While our bioinformatics approach has provided significant insights into potential key players in DR pathogenesis, we recognize that the identification of ALKBH, ATP13A2, and PSIP1 as potential biomarkers or therapeutic targets is preliminary and necessitates further experimental validation.

In conclusion, our study marks progress in molecular biology and disease research through the application of technologies such as AlphaFold 2, single-cell sequencing, machine learning and NMF. This methodological synergy has not only enriched our understanding of the molecular landscape of PDR but also highlighted the importance of ORGs in its pathogenesis. Our research underscores the value of harnessing advanced technologies to explore disease mechanisms and therapeutic innovations.

## Data availability statement

The original contributions presented in the study are included in the article/**Supplementary Materials**, further inquiries can be directed to the corresponding author/s.

## Ethics statement

Ethical approval was not required for the study involving humans in accordance with the local legislation and institutional requirements. Written informed consent to participate in this study was not required from the participants or the participants’ legal guardians/next of kin in accordance with the national legislation and the institutional requirements.

## Author contributions

JW: Writing – original draft, Writing – review & editing. HS: Writing – original draft, Writing – review & editing. LM: Formal analysis, Writing – original draft. YL: Formal analysis, Writing – original draft. ZW: Formal analysis, Writing – original draft. ZP: Conceptualization, Writing – review & editing. M-MY: Methodology, Writing – review & editing.
